# Intravenous Infusion of High Dose Selenite in End-Stage Cancer Patients: Analysis of Systemic Exposure to Selenite and Seleno-Metabolites

**DOI:** 10.3390/biomedicines11020295

**Published:** 2023-01-20

**Authors:** Olof Breuer, Ola Brodin, Ali Razaghi, David Brodin, Bente Gammelgaard, Mikael Björnstedt

**Affiliations:** 1 Department of Laboratory Medicine, Division of Clinical Pharmacology, Karolinska Institutet, Karolinska University Hospital-Huddinge, SE-14186 Stockholm, Sweden; 2 Department of Laboratory Medicine, Division of Pathology, Karolinska Institutet, Karolinska University Hospital-Huddinge, SE-14186 Stockholm, Sweden; 3 Tema Cancer, Karolinska University Hospital-Solna, SE-14186, Stockholm, Sweden; 4 Department of Biosciences and Nutrition, Karolinska Institutet, SE-14183 Huddinge, Sweden; 5 Department of Pharmacy, Faculty of Health and Medical Sciences, University of Copenhagen, Universitetsparken 2, 2100 Copenhagen, Denmark

**Keywords:** selenotherapy, selenosugar, cancer, selenite, selenium

## Abstract

Cancer is one of the main causes of human death globally and novel chemotherapeutics are desperately required. As a simple selenium oxide, selenite is a very promising chemotherapeutic because of pronounced its dose-dependent tumor-specific cytotoxicity. We previously published a first-in-man systematic phase I clinical trial in patients with cancer (from IV to end-stage) (the SECAR trial) showing that selenite is safe and tolerable with an unexpectable high maximum tolerated dose (MTD) and short half-life. In the present study, we analyzed the selenium species in plasma samples, from the patients participating in the SECAR trial and from various time points and dose cohorts using LC-ICP-MS. In conclusion, selenite, selenosugars, and 1–2 unidentified peaks that did not correspond to any standard, herein denoted ui-selenium, were detected in the plasma. However, trimethylated selenium (trimethylselenonoium) was not detected. The unidentified ui-selenium was eluting close to the selenium-containing amino acids (selenomethionine and selenocysteine) but was not part of a protein fraction. Our data demonstrate that the major metabolite detected was selenosugar. Furthermore, the identification of selenite even long after the administration is remarkable and unexpected. The kinetic analysis did not support that dosing per the body surface area would reduce interindividual variability of the systemic exposure in terms of trough concentrations.

## 1. Introduction

Selenium is an antioxidant at nutritional levels; many publications report growth-inhibitory and cytotoxic effects of redox-active selenium compounds in from moderate to high concentrations against cancer [1,2,3]. Selenite, the most widely studied selenium species, is an inorganic selenium salt that is highly reactive and metabolized already in the gastric mucosa cells due to a reaction with reduced glutathione [3]. Furthermore, selenite is an excellent substrate for mammalian Thioredoxin reductase (TrxR) with Km, Vmax, and Kcat numbers close to the natural substrate Thioredoxin (Trx). Under anaerobic conditions, the reaction between selenite and TrxR stops after the consumption of three molecules of NADPH, showing that selenite is reduced to the highly reactive selenide. As soon as oxygen is admitted to the system, a linear reaction is reassumed. Selenide efficiently redox cycles with oxygen, producing reactive oxygen species (ROS) until the system is exhausted of thiols and/or NADPH [3,4]. The non-stoichiometric production of ROS and the resulting oxidative stress is a major mechanism of the growth inhibitory and antineoplastic effects of selenite, thus founding the basis for the application of selenite as a chemotherapeutic agent against cancer [3,5]. For instance, many publications show that tumor cells are more sensitive compared to normal cells [6,7,8]. Additionally, selenite is more efficiently assimilated and accumulated in highly drug-resistant neoplastic cells [1,4]. The higher sensitivity together with the more efficient uptake by tumor cells is explained by the resistant phenotype expressed by cancer cells of which a characteristic feature is a higher level of thiols intracellularly, increased reducing the extracellular environment, and efficient mechanisms to withhold a reducing extracellular milieu. Of particular interest are the MRP family of proteins and the Cystine/Glutamate (Xct) antiporter, both known to convey drug resistance [3,7]. We have previously shown that these factors potently increase selenite uptake using extracellular reduction mediated by cysteine that is constantly supplied in the extracellular space through the Xct-antiporter and MRPs [9].

Selenide is crucial for the specific incorporation of selenocysteine into selenoproteins. Furthermore, selenide may undergo methylation to methylselenol and dimethyldiselenide, which is volatile and causes the well-known signs of selenium overload, garlic odor in breath, and trimethylselenonium that is excreted in urine. The leading urinary excretory metabolite, however, is the selenosugar, *Se*-methylseleno-*N*-acetylgalactosamine (SeGal) [3].

Due to the high reactivity with thiols, selenite may not be detected in the plasma after oral administration. To study the pharmacology of selenite and monitor any effect, intravenous administration is required. Previously, selenite has not been identified in plasma. In contrast, we reported that selenite could be detected in plasma using LC-ICP-MS after intravenous infusions [10].

In an early clinical study on high doses per oral sodium selenite as an adjunct to radiotherapy in prostate cancer, one conclusion was that pharmacokinetic studies are important to find optimal treatment schedules [11]. In 2015, we published the first-in-man phase I clinical trial in patients with end-stage malignancies. The tolerance was unexpectedly high with a maximal tolerable dose of 10.2 mg/m^2^ body surface. However, the half-life was short (18.5 h) and there were no signs of selenosis. Antineoplastic effects have been reported to peak after 48 h of exposure explaining why the observed effects were relatively sparse [12].

To study the metabolism of high-dose selenite in cancer patients, we analyzed the plasma samples from various time points and dose cohorts using LC-ICP-MS and studied the kinetic parameters. The purpose of the present study was thus to explore which metabolites appeared after a high dose of intravenous selenite infusion and how long the active drug selenite could be traced in the plasma samples. In addition, the relationships between systemic exposures and tumor growth inhibition were explored.

## 2. Materials and Methods

### 2.1. Clinical Samples

The clinical samples were obtained from the previously published SECAR study (Table 1) [12]. Three patients were included in each cohort except the highest which included 6 patients. In total, 10 cohorts were needed before the maximum tolerable dose could be defined. The starting dose was 0.5 mg/m^2^ body surface and, for the first 4 cohorts, the dose was escalated by 0.5 mg/m^2^ body surface. The plasma samples were extracted before the start of selenite treatment. After finishing the fourth cohort without any dose-limiting toxicity an amendment to the Ethical Committee and the Medical Product Agency was approved (see Institutional Review Board Statement) allowing dose escalations of 50% between cohorts and the inclusion of other malignancies except lung carcinoma. The protocol for the first four cohorts included two periods of 10 days of infusion separated by a week of rest. However, from the fifth cohort onwards, the third and fourth treatment weeks and the treatment-free week were omitted and thus the patients received only 10 treatments. Dosing was not performed during weekends. Both groups were followed by chemotherapy of the same formulation that the patients before inclusion developed resistance to. The evaluation of the systemic exposure to the parent compound selenite and seleno-metabolites following the first 2 weeks of dosing at dose levels of from 1.0 up to 15.3 mg/m^2^ is reported here. The tumor status according to RECIST was measured using a CT-scan just before and after the selenite treatment. The elapsed time between CT-scans was approximately 7 weeks in the first 13 patients and 4 weeks for the remaining patients.

### 2.2. Sampling Schedule

The dosing regimen was modified during the study such that doses of 3 mg/m^2^ and higher were only administered during two consecutive 7-day cycles, each consisting of 5 consecutive days of daily selenite infusions. Hence, to evaluate the dose-systemic exposure relationship across the entire dose range of the study, three sampling occasions were selected that were considered comparable between all applied dosing regimens; baseline (before the 1st dose was administered), trough value at predose Day 5 (Week 1, 96 h after start), and trough value at predose Day 12 (Week 2, 264 h after the start).

### 2.3. Seleno-Metabolite Analysis

The plasma samples (200 µL) were transferred to Vivaspin centrifugal filter units (Sartorius) with semi-permeable membranes of molecular weight cut-off 3000 Da, centrifuged at 14,000× *g* for 30 min, and the filtrate was then transferred to LC vials and analyzed using LC-ICPMS. Isocratic chromatography was performed on a Gemini C18 column (250 × 2 mm), with 5 µm (Phenomenex) in a mobile phase consisting of 200 mM ammonium acetate in 5% methanol, pH (6.7) with a flow rate of 0.2 mL/min, and an injection volume of 10 µL. The LC-system (Agilent 1100 System) was hyphenated to a Sciex ELAN DRCe ICPMS system (Perkin Elmer) and the isotopes ^77^Se, ^80^Se, and ^82^Se were monitored. The quantitation was performed using post-column isotope dilution analysis. An enriched ^77^Se solution in the mobile phase was continuously introduced and the post-column with a syringe pump connected to the eluent flow of the LC and the three Se isotopes were monitored. The isotope ratios were calculated, mass flow chromatograms were obtained after the application of the isotope dilution equation to each data point, and peak areas were calculated using the OriginPro software. The linearity was established for selenite and selenosugar and the precision was better than 9% in the plasma samples. Based on a certified selenite standard, the accuracy was 99.5% and LOQ was 1.3 µg/L. All the details are previously described [10].

## 3. Results

### 3.1. Baseline Levels of Seleno-Metabolites in Plasma

Only selenite and total selenium were consistently quantifiable at baseline with a LOQ of 1.3 µg/L. The baseline plasma concentrations of selenite, selenosugar, total selenium, and ui-selenium are listed in Table 2.

### 3.2. Dose-Plasma Concentration of Seleno-Metabolites

The samples at Week 1 or Week 2 contained selenite, selenosugar, and in some cases small amounts of one–two unidentified selenium compounds (ui-selenium). These unidentified compounds were eluting close to the selenium-containing amino acids (selenomethionine and selenocysteine) but were not co-eluting with any available standard. As the samples were exposed to ultrafiltration before analysis, the unknown species were not proteins.

**Figure 1:**
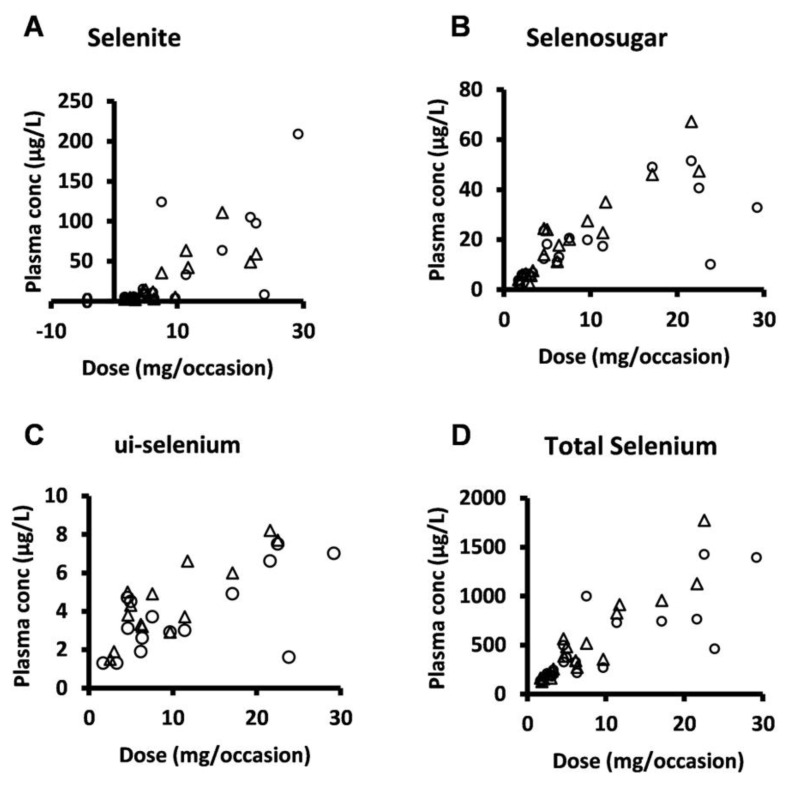
Plasma concentration-dose relationship for selenite and seleno-metabolites at 24 h after dose (i.e., trough value) following four consecutive daily intravenous infusions of selenite during Week 1 (circles) and Week 2 (triangles). (**A**) selenite, (**B**) selenosugar, (**C**) ui-selenium, (**D**), total selenium.

**Figure 2:**
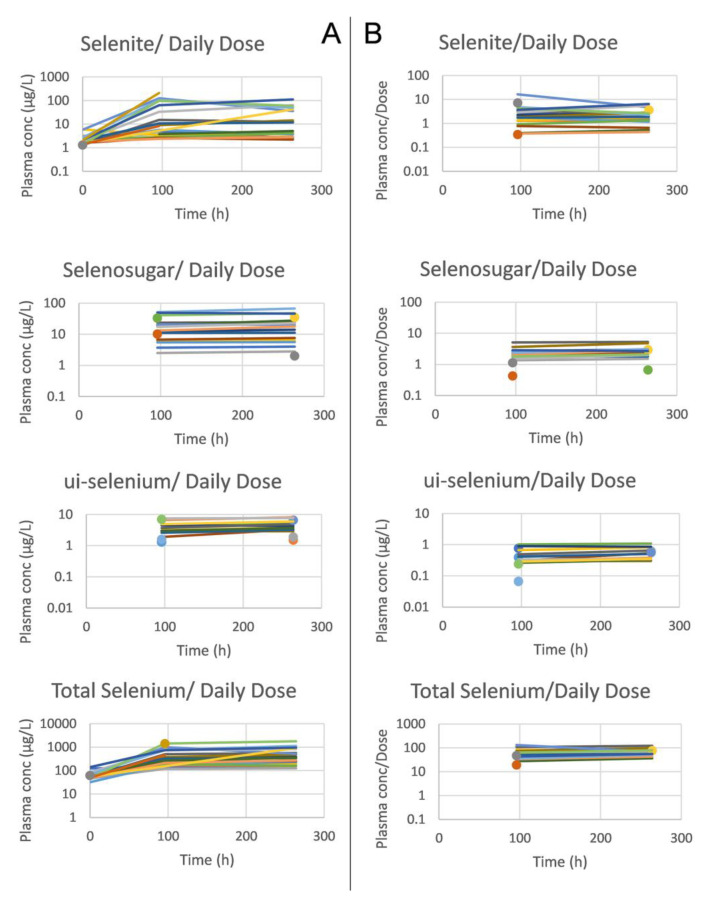
Mean trough concentrations per subject of selenite, selenosugar, ui-selenium and total selenium in plasma (from upper to lower panels), and before (**A**) and after (**B**) dose normalization. The samples were extracted at baseline and 24 h after dose (i.e., trough value) following four consecutive daily intravenous infusions of selenite during Week 1 and Week 2. Single observations are marked with a circle (Week 1) and triangle (Week 2).

Selenosugar and ui-selenium became quantifiable in the trough plasma samples upon infusion of selenite for 4 consecutive days (Table 2). In both Week 1 and 2, the statistically significant relationships were seen between the amount of selenite administered per occasion to each patient and the trough plasma concentrations for selenite (Pearson’s correlation coefficient r = 0.80, *p* < 0.001, Week 2), ui-selenium (r = 0.85, *p* < 0.001, Week 2), total selenium (r = 0.93, *p* < 0.001, Week 2), and selenosugar (r = 0.94, *p* < 0.001, week 2) (Figure 1). Consistent with this result, the normalization of the plasma concentration of these compounds with respect to the dose decreased the variability in terms of the relative SD for selenite, total selenium, and selenosugar (Table 2 and Table 3 and Figure 2), but less so for ui-selenium. In addition, there was only a minor fluctuation between Week 1 and Week 2 in the dose-normalized plasma concentrations indicating that a pharmacokinetic steady-state had been reached within 5 days after the start of treatment (Figure 2).

### 3.3. Systemic Exposure of Selenite in Relation to Patient-Specific Variables

**Figure 3:**
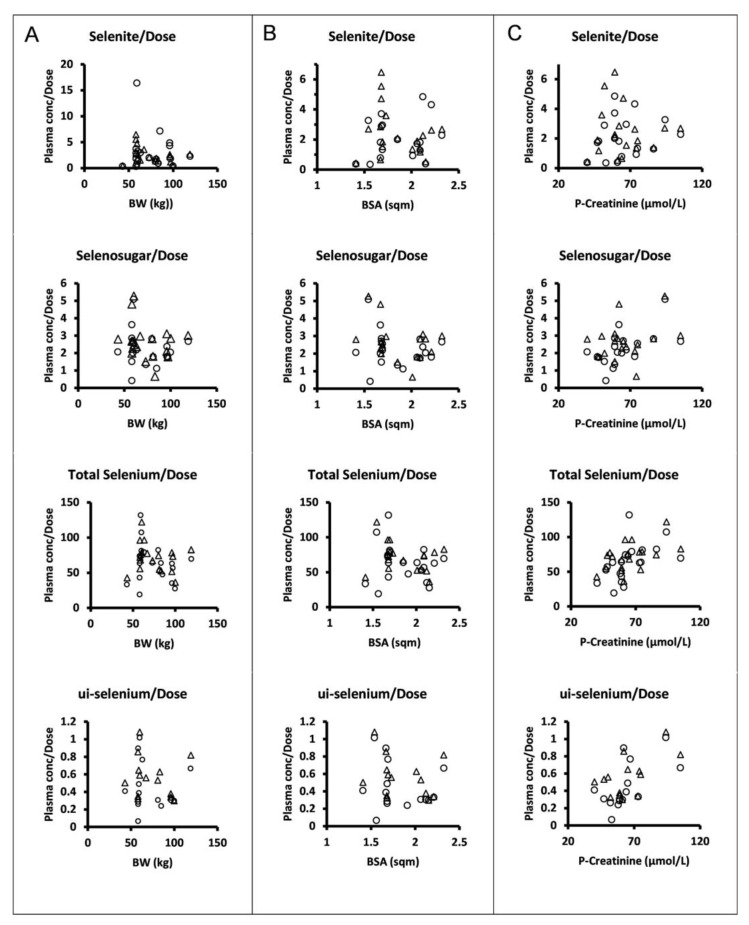
Dose normalized mean trough concentrations of selenite, selenosugar, total selenium, and ui-selenium in plasma (from upper to lower panels) at 24 h after dose following four consecutive daily intravenous infusions of selenite during Week 1 (circles) and Week 2 (triangles) versus individual body weight (**A**), body surface area (**B**), and plasma creatinine (**C**). Normalization was performed with respect to the amount of selenite administered per patient on each dosing occasion.

The dose levels were chosen and escalation increments were performed in relation to body surface area. However, there was no apparent relationship between the plasma concentrations normalized to the amount of selenite administered per occasion and body surface area or body weight for selenite, selenosugar, total selenium, or ui-selenium (Figure 3).

### 3.4. Selenite Concentration and Tumor-Growth Inhibition

**Figure 4:**
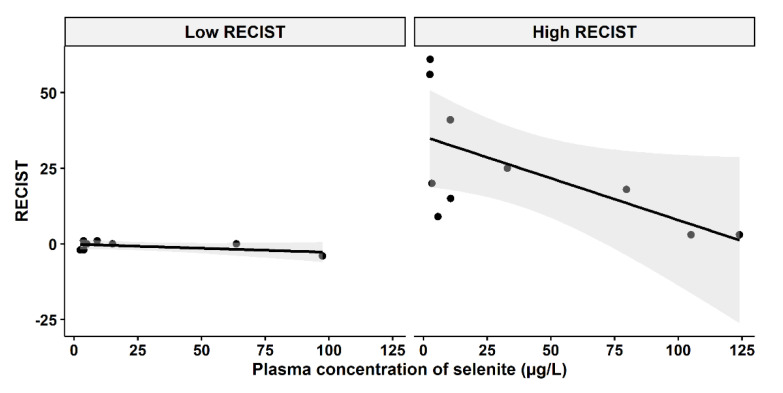
Relationship between RECIST and systemic exposure to selenite. Left panel: Low RECIST-group (RECIST ≤ 1 mm), Pearson two-sided correlation coefficient, r = −0.5467, *p* = 0.1609. Right panel: High RECIST group (RECIST > 1 mm), r = −0.6239, *p* = 0.05391. The shadowing in grey represents the confidence interval.

A question of clinical interest is if higher plasma concentrations of selenite might increase the responses of carcinomas. An indication of a relationship between higher plasma concentration and less cancer growth and even shrinkage was found, depending on the cut-off level of growth inhibition according to RECIST evaluation before and after the selenite treatment (Figure 4). The tumor population was divided into two groups (low-RECIST and high-RECIST) with a cut-off value of 1 mm tumor growth (RECIST). The cut-off limit, 1 mm, was chosen since it might be considered to be clinically relevant.

## 4. Discussion

Cancer is one of the main causes of human death globally and, despite great progress in the prognosis of some malignancies, the prognosis remains very poor for highly resistant visceral malignancies. Hence, novel chemotherapeutics are desperately required. Redox-active selenium compounds, including selenite, have remarkable tumor-specific cytotoxic properties [12]. This effect is particularly pronounced in cells that have developed resistance to cytostatic drugs. Resistant tumor cells accumulate selenium due to the facilitated uptake by transporters conferring drug resistance, including the Xct-antiporter and the MRP-superfamily of proteins [9]. Selenium has, despite this, a great body of preclinical data not yet introduced to the routine care of cancer patients due to the lack of human clinical trials. In 2015, we published a first-in-man systematic phase I clinical trial (the SECAR study) in patients with cancer (from IV to end-stage) showing that sodium selenite is safe and tolerable if 10.2 mg.m^2^ MTD or less is administered [12]. The metabolic faith of selenite in humans after repeated high-dose iv infusions has so far been unknown. The available data originate at large from animal studies and not from human subjects.

After the reduction in selenite enzymatically by redox enzymes or chemically by low molecular or protein-bound thiols, the anionic highly reactive species selenide is formed [4]. Selenide is readily absorbed by cells and the extracellular reduction in selenite facilitates the selenium uptake. The reduced extracellular environment surrounding highly resistant cells, as a characteristic of the resistant phenotype, may by this mechanism explain the selective and marked tumor-specific cytotoxicity of selenite [9]. Selenide is assumed to be not only the common intermediate for all Se nutritional sources but also the checkpoint metabolite for Se utilization or excretion and, not least, methylation reactions. Se absorbed by the body within the normal nutritional Se intake range is suggested to be mostly excreted in urine as selenosugar, 1-methylseleno-N-acetyl-d-galactosamine, in mammals. Selenosugar was the main metabolite in our study, although high supranutritional levels were used. An excessive amount of selenium is methylated to mono-di and trimethylated forms and the leading methylated urinary species is trimethylselenonium [13]. Trimethylselenonium increases with excessive selenium intake or body burden, suggesting that trimethylselenonium may serve as a urinary biomarker for both excessive selenium intake and body burden as well as a toxic dose of selenium [13]. However, most studies concerning the formation of trimethylselenonium have been performed using oral regimens. In addition, excess selenium intake may lead to the synthesis of several methylated species including volatile species, e.g., Dimethyldiselenide. The methylated volatile species will cause a distinct garlic odor in the breath. In the SECAR trial, the research nurses noted a garlic odor in the breath of a few patients. This observation indicates that volatile forms were synthesized and that one excretion pathway was from the breath in addition to urine. Despite high levels of selenite administered, trimethylselenonium was not detected in any of the cohorts investigated.

Selenoprotein P (SELENOP) is a proven biomarker of Se status [14]. Following cleavage of the signal peptide, SELENOP is mostly produced in the liver and released into the plasma. SELENOP performs two distinct tasks: it has GPX-like activity to reduce phospholipid hydroperoxide and Se transport activity to deliver Se to cells. In addition to maintaining selenoenzymes in various tissues, SELENOP is essential for Se metabolism and antioxidative defense [15]. We already hypothesized that SELENOP may reflect selenium levels in therapeutic dosages of selenite for clinical applications. For this purpose, blood samples from the same patients in the SECAR clinical trial phase I were used. The total Se was quantified using spectroscopy and SELENOP was validated using ELISA. SELENOP was increased in high-dosage selenite infusions. Thus, we concluded that circulating SELENOP is a suitable biomarker for therapeutic applications of selenite in upper intake levels [16]. Following our previous result of SELENOP, therefore, we can hypothesize that exceeding levels of selenite have been invested for SELENOP production.

Our data do not support that dosing per body surface area would reduce the interindividual variability of systemic exposure in terms of trough concentrations, since the normalization of the parent compound or its seleno-metabolites to the amount of selenite administered per dosing occasion did not show any relationship with the body surface area. However, one can discern statistically significant correlations between the plasma creatinine concentration and dose-normalized total selenium (r = 0.52, *p* < 0.05, Week 1; r = 0.53, *p* < 0.05, Week 2), ui-selenium (r = 0.61, *p* < 0.05, Week 1; r = 0.60, *p* < 0.05, Week 2), and selenosugar (r = 0.58, *p* < 0.01, Week 1; not significant Week 2). This might support that renal excretion is a contributing path to the elimination of selenium-containing compounds [17].

The lack of a strong correlation between higher plasma concentrations of selenite and cancer growth inhibition might have different explanations. The study was not designed, or dimensioned, to prove any efficacy. The carcinomas were clinically very advanced and heterogenous with both fast- and slow-growing tumors of different histology. Furthermore, this was a phase I trial in which the toxicity, safety, and kinetics were the primary and secondary endpoints, whereas the pharmacodynamic response was only included as an exploratory endpoint.

Even if the production of free oxygen radicals (ROS) is the primary mechanism for cancer cell killing, selenite also changes the redox balance of cells, which has an impact on many cellular functions such as proliferation, apoptosis, and the expression and function of membrane pumps [3]. It has also been demonstrated that high-dose selenite can inhibit the expression of cancer-stimulating genes and decrease the expression of different membrane receptors such as androgen receptors in prostate carcinoma [18] and exert immune-activation effects [1] even though the effect on soluble PDL-1 is unclear [19]. Concerning ROS production in vitro, the studies demonstrate that higher concentrations of selenite increase the effect [3] and it is reasonable to suspect that the same is also true in the clinical situation.

The concentration of selenite has been proven to be critical for effect and the dose may vary depending on the cell type [4]. Low concentrations might even stimulate tumor growth as demonstrated by several studies. Selenium is an essential part of cell culture media for serum-free conditions [4]. Most cancer cells are sensitive to from moderate to high concentrations in the span of 5–15 µM [3,4]. In fact, 15–30 µM proved to be exceptionally efficient in an ex vivo tissue culture of human pancreatic ductal adenocarcinoma with a marked specificity to tumor cell cytotoxicity and had a limited or no effect on ambient tissues [20]. The exposure time is also important and, in laboratory experiments, the maximum effect is usually observed after 48 h of exposure—a factor that might be one explanation for the lack of tumor response in the SECAR trial.

According to Figure 4, there might be two populations of tumor growth responses, whereof one consists of either slowly growing tumors or tumor growth inhibited by other means, i.e., immune activation. An example of the latter is the observation in the lower left part of Figure 4, where a patient had a tumor that neither grew nor shrank after the selenite treatment, but later regressed and after half a year had disappeared and never recurred. The tumors in the right panel of Figure 4, on the other hand, might depend on ROS production for growth inhibition. It should be noted that this is a hypothesis and our results in this study do not confirm an association between higher concentration and tumor regression.

The SECAR trial is to our knowledge the first published study where high doses of selenite were administered intravenously to advanced cancer patients. The speciation analysis showing the prolonged presence of selenite together with high tolerance (MTD 10.2 mg/m^2^) and short half-life of total selenium demonstrates the potential to safely use iv administration of selenite in patients [12]. Even though this phase I study did not show any significant effect on the tumor burden, the results merit further investigations of the potential of selenium in the treatment of cancer.

## 5. Conclusions

The major selenium metabolite in plasma after repeated iv administrations of selenite was selenosugar. Furthermore, selenite was detected in plasma even a long time after administration. We conclude that selenite dosing per body surface area would not reduce the interindividual variability of systemic exposure in terms of trough concentrations. However, for future studies and applications of selenite, the MTD must be considered, as an adequate guide for dosing.

## Figures and Tables

**Table 1:** Characteristics of patients. Total dose calculated according to body surface area. ASAT (aspartate-aminotransferase) with normal value (76≤). Plasma-creatinine with normal value (100≤). **Abbreviations:** Met: metastatic disease and L: locally advanced disease.

Age	Tumor Histology	Dose/ m^2^ (mg/m^2^)	Total Dose (mg)	Stage	ASAT (µkat/L)	P-Creatinine (µM)
62	Squamous cell carcinoma lung	1	1.7	L	0.42	61
73	Adenocarcinoma, lung	1	2.1	Met	0.46	106
76	Squamous cell carcinoma, lung	1	1.8	Met	0.29	52
58	Non-small cell lung cancer	1.5	2.5	Met	1.49	65
58	Squamous cell, lung cancer	1.5	3.1	Met	0.51	38
37	Adenocarcinoma, lung	1.5	3	Met	0.40	65
67	Small-cell lung cancer	2	4.6	Met	0.57	84
62	Large cell lung cancer	2	3.4	Met	0.30	51
75	Adenocarcinoma, lung	3	4.8	L	0.38	87
45	Adenocarcinoma, lung	3	5	Met	0.22	67
59	Adenocarcinoma, lung	3	6.1	Met	0.60	81
65	Squamous cell carcinoma, lung	4.5	9.8	L	1.10	65
60	Adenocarcinoma, lung	4.5	7.4	Met	0.81	54
65	Adenocarcinoma, lung	4.5	6.2	Met	0.32	37
46	Colon carcinoma	6.8	11.5	Met	0.66	54
61	Colon carcinoma	10.2	22.5	Met	1.21	85
62	Non-small cell lung cancer	10.2	17.6	Met	0.37	61
53	Colon carcinoma	15.3	29	Met	1.85	58
65	Malignant mesothelioma	15.3	32.6	L	0.25	70
41	Adenocarcinoma, lung	12.8	23.9	Met	0.45	58

**Table 2:** Mean trough concentrations of selenite and seleno-metabolites in plasma at baseline and 24 h after dose following four consecutive daily intravenous infusions of selenite during Week 1 and Week 2. Mean, SD, and range are tabulated across the entire dose range of the study.

Analyte	Time (week)	Mean Conc. (µg/L)	N	SD	Relative SD	Range
Selenite	Baseline	2.34	20	1.31	0.57	<LOQ-6.00
	1	35.9	20	56	1.56	2.40–209
	2	23.4	19	29.6	1.27	2.20–111
Selenosugar	Baseline	-	0	-	-	All < LOQ
	1	18.5	19	15	0.81	<LOQ-51.5
	2	20.6	19	17.8	0.86	2.0–67
Total selenium	Baseline	68.4	21	25.1	0.37	32–140
	1	488	20	399	0.82	119–1425
	2	516	19	430	0.83	125–1774
ui-selenium	Baseline	-	0	-	-	All < LOQ
	1	3.77	15	2.04	0.54	<LOQ-7.5
	2	4.05	14	2.03	0.45	<LOQ-8.2

**Table 3:** Dose normalized mean trough concentrations of selenite and seleno-metabolites in plasma before the 5th and 10th treatments, 24 h after respective previous infusion. Normalization was performed with respect to the amount administered on each dosing occasion per individual. Dose normalized mean, SD, and range are tabulated across the entire dose range of the study.

Analyte	Time (week)	Mean Conc/Dose (µg/L)	N	SD	Relative SD	Range
Selenite	1	3.03	20	3.58	1.18	0.35–16.4
	2	2.45	19	1.65	0.68	0.44–6.48
Selenosugar	1	2.25	19	1.00	0.44	<LOQ-5.09
	2	2.63	19	1.05	0.40	0.66–5.28
Total selenium	1	63.5	20	26.7	0.42	19.3–132
	2	71.6	19	20.3	0.28	36.7–122
ui-selenium	1	0.44	15	0.26	0.60	<LOQ-1.017
	2	0.56	14	0.23	0.41	<LOQ-1.082
